# Influence of insecticides flubendiamide and spinosad on biological activities in tropical black and red clay soils

**DOI:** 10.1007/s13205-013-0188-3

**Published:** 2013-12-18

**Authors:** G. Jaffer Mohiddin, M. Srinivasulu, K. Subramanyam, M. Madakka, D. Meghana, V. Rangaswamy

**Affiliations:** 1 Department of Microbiology, Sri Krishnadevaraya University, Anantapur, 515 055 Andhra Pradesh India; 2 Department of Biotechnology and Bioinformatics, Yogi Vemana University, Kadapa, 516 003 Andhra Pradesh India; 3 Plant Molecular Biology Unit, Department of Biotechnology and Genetic Engineering, Bharathidasan University, Tiruchirappalli, 620024 Tamil Nadu India; 4 Radioactive Waste Management (Bioremediation) Lab, Division of Advanced Nuclear Engineering, Pohang University of Science and Technology, Pohang-si, Republic of Korea; 5 Present Address: Department of Life Sciences and Agriculture, Universidad de las Fuezas Armada, Sangolqui, Quito, Ecuador South America

**Keywords:** Enzyme activities, Flubendiamide, Groundnut (*Arachis hypogaea* L.) soils, Spinosad

## Abstract

A laboratory experiment has been conducted to investigate the ecological toxicity of flubendiamide and spinosad at their recommended field rates and higher rates (1.0, 2.5, 5.0, 7.5, 10.0 kg ha^−1^) on cellulase, invertase and amylase in black and red clay soils after 10, 20, 30 and 40-day exposure under controlled conditions in groundnut (*Arachis hypogaea* L.) soils of Anantapur District, Andhra Pradesh, India. Flubendiamide and spinosad were stimulatory to the activities of cellulase, invertase and amylase at lower concentrations at 10-day interval. The striking stimulation in soil enzyme activities noticed at 2.5 kg ha^−1^, persists for 20 days in both soils. Overall, the higher concentrations (5.0–10.0 kg ha^−1^) of flubendiamide, and spinosad were toxic or innocuous to cellulase, invertase and amylase activities, respectively. The results of the present study thus, clearly, indicate that application of the insecticides in cultivation of groundnut, at field application rates improved the activities of cellulase, invertase and amylase in soils.

## Introduction

In modern agriculture, it has become a common trend to apply different groups of pesticides, either simultaneously or in succession, for effective control of a variety of pests (Quazi et al. 2011). Pesticides are deliberately introduced into agricultural systems with various formulations to protect crops against weeds, insects, fungi and other pests (Yang et al. 2007; Moorman 1989; Singh et al. 1999; Bhuyan et al. 1992; Chu et al. 2008). However, much of the applied pesticides will finally reach the soil often leading to a combined contamination of pesticide residues in the soil environment (Chu et al. 2008), which may affect the growth and activity of soil microbial communities (Singh and Singh 2005), and in turn affect the enzyme activities. Increasing use of pesticides in agriculture led to the development of soil microbial testing programme for examination of the side effects (Swaminathan et al. 2009). The testing programmes include measurement of activities of soil enzymes, and physicochemical properties.

The economy of India is largely dependent on agricultural production. Better harvest requires rigorous cultivation, irrigation, fertilizers and pesticides to protect plants from pests and plant diseases. In India, 15–20 % of all produce is destroyed by pests (Bhalerao and Puranik 2009). Groundnut (*Arachis hypogaea* L.) is one of the most important cash crops grown in Indian agricultural soils with the highest yield among the oil seeds’ crops (Singh and Singh 2005; Menon et al. 2004; Bera et al. 2002) and is the primary source of edible oil in India (Ramesh babu et al. 2002). India is a world leader in groundnut farming, with 6.0 million hectares of the cultivated area during the year 2010–11 (USDA 2011). Within India, Andhra Pradesh State ranks first in area and production (Hegde and Kiresur 1999). Among different regions of Andhra Pradesh, Anantapur District, a semi-arid region relies on groundnut cultivation, predominantly (Anonymous 2011). In spite of its high range of cultivation, groundnut productivity is low, fluctuating around 9 q/ha on average, and an annual yield loss of Rs. 150 crores due to pests has been reported (Loganathan et al. 2002). Among the pesticides, insecticides of flubendiamide and spinosad are in the current list of modern pesticides in Indian agriculture used to control incidence of pest attack over groundnut crop.

Soil is a natural system containing microbes which are the driving force behind many soil processes, including transformation of organic matter, nutrient release and degradation of xenobiotics (Zabaloy et al. 2008). Many studies have shown that biological parameters have been used to assess soil quality and health as affected by agricultural practices (Gianfreda et al. 2005; Truu et al. 2008; Garcia-Ruiz, et al. 2009). In this respect, soil enzymes can be used as potential indicators of soil quality for sustainable management because they are sensitive to ecological stress and land management practices (Tejada 2009). Flubendiamide represents a novel class of insecticides with extremely high activity against a broad spectrum of lepidopterous insects (Tohnishi et al. 2005). Spinosad is a biologically derived insecticide that consists of two active compounds, spinosyns A and D, produced by fermentation culture of an actinomycete isolated from soil (*Saccharopolyspora spinosa* Mertz and Yao). Structurally, these compounds are macrolides and contain a unique tetracycling system to which two different sugars are attached (Kirst et al. 1992). Negative impact of pesticides on soil enzymes activities has been widely reported throughout the literature (Ismail et al. 1998; Menon et al. 2005) unfortunately no reports were available on these two new insecticides on enzyme activities.

The quorum-sensing systems allow bacteria to monitor their environment for the presence of other bacteria and to respond to fluctuations in the number and/or species present by altering particular behaviors. Most quorum-sensing systems are species- or group-specific, which presumably prevents confusion in mixed-species environments. However, some quorum-sensing circuits control behaviors that involve interactions among bacterial species. These quorum-sensing circuits can involve both intra- and interspecies communication mechanisms. Finally, anti-quorum-sensing strategies are present in both bacteria and eukaryotes and these are apparently designed to combat bacteria that rely on cell-cell communication and for the successful adaptation to particular niches. Many enzymes of both microbial or plant origins have been recognized to be able to transform pollutants at a detectable rate and potentially suitable to restore polluted environment. The main enzymatic classes involved in such a process are hydrolases, dehalogenases, and oxidoreductases. Amide, ester and peptidic bonds undergo hydrolytic cleavage by amidases, esterases and proteases in several xenobiotic compounds and may lead to products with little or no toxicity. Hydrolases responsible for the cleavage of pesticides are among the best studied groups of enzymes. Most of these hydrolases are extracellular enzymes, except for the cell wall-bound enzymes of penicillium and arthrobacter sp., which hydrolyze barban and propham.

Cellulase can catalyze hydrolysis of 1, 4, beta- d-glycosidic bonds of cellulose and is also an important indicator for carbon circulation. Invertase is known to be a very stable and persistent enzyme, and its association with soil components is well documented (Kiss et al. 1978). Amylase plays an important role in biochemical reactions and nutrient cycling. Apparently, it has become necessary to determine the effects of agronomically needed pesticides (flubendiamide and spinosad), applied at recommended levels and at higher doses, in order to establish the significance, in terms of biogeochemical reactions and nutrient cycling. Hence the present study was carried out to determine the influence of insecticides on the activity of cellulase, invertase and amylase in two groundnut soils of Anantapur district, Andhra Pradesh, India from December 2, 2010 to July 15, 2011.

## Materials and methods

### Soils

Black and red clay soils were used in the present study. Soil samples taken from groundnut-cultivated fields of Anantapur district, Andhra Pradesh, India, were chosen with a known history of pesticides use, from a depth of 12 cm, air-dried and sieved through 2 mm sieve before usage. Mineral matter of soil samples such as sand, silt, and clay contents were analyzed with use of different sizes of sieves by following the method of Alexander (1961). Cent percent water-holding capacity of soil samples was measured by finding amount of distilled water added to both the soil samples to get saturation point and then 60 % water-holding capacity of soil was calculated by the Johnson and Ulrich method (1960). Soil pH was measured at 1:1.25 soil-to-water ratio in a Systronics digital pH meter with calomel glass electrode assembly. Organic carbon content in soil samples was estimated by Walkley–Black method, and the organic matter was calculated by multiplying the values with 1.72 (Jackson 1971). Electrical conductivity of soil samples after addition of 100 ml distilled water to 1 g soil samples was measured by a conductivity bridge. Total nitrogen content in soil samples was determined by the method of micro-Kjeldahl method (Jackson 1971). Content of inorganic ammonium–nitrogen in soil samples after extraction of 1 M KCl by Nesslerization method (Jackson 1971), contents of nitrite–nitrogen (Barnes and Folkard 1951) and contents of nitrate–nitrogen by Brucine method (Ranney and Bartlett 1972) after extraction with water were determined, respectively. Physicochemical characteristics of the two soils are listed in Table 1.

**Table 1:** Physicochemical properties of the soils

Properties	Black clay soil	Red clay soil
Sand (%)	68.45	53.25
Silt (%)	21.45	27.12
Clay (%)	10.0	19.8
pH^a^	7.8	6.7
Water holding capacity (ml g^−1^ soil)	0.7	0.4
Electrical conductivity (mmhos)	258	232
Organic matter (%)^b^	1.34	0.74
Total nitrogen (%)^c^	0.086	0.038
NH_4_^+^–N (μg g^−1^ soil)^d^	6.96	6.01
NO_2_^−^–N (μg g^−1^ soil)^e^	0.58	0.42
NO_3_^−^–N (μg g^−1^ soil)^f^	0.94	0.73

^a^1:1.25 = soil:water slurry

^b^Walkley–Black method (Johnson and Ulrich 1960)

^c^Micro-Kjeldahl method (Johnson and Ulrich 1960)

^d^Nesslerization method (Johnson and Ulrich 1960)

^e^Diazotization method (Ranney and Bartlett 1972)

^f^Brucine method (Barnes and Folkard 1951)

### Insecticides

In order to determine the influence of selected insecticides on the microbial activities, commercial grades of flubendiamide and spinosad were obtained from Bayer’s Science India.

### Soil treatment

The soil ecosystem stimulating non-flooded portions of the soil samples were added in test tubes (25 × 150 mm) and moistened with water in order to maintain at 60 % water-holding capacity. Same model was used previously to elucidate the effect of insecticides on microbial activities by Mohiddin et al. (2011).

### Cellulase (EC 3.2.1.4), invertase activity (EC 3.2.1.26) and amylase activity (EC 3.2.1.1)

Five-gram portion of the soil samples was weighed and dispersed into sterile test tubes (25 × 150 mm). Stock solutions from selected insecticides were added to the rate of 10, 25, 50, 75 and 100 μg g^−1^ soil equivalent to field application rates of 1.0, 2.5, 5.0, 7.5 and 10.0 kg ha^−1^ respectively. Soil samples without insecticide treatment served as controls. Soil samples were mixed thoroughly for uniform distribution of insecticide that was added. Triplicates were maintained for each treatment at room temperature (28 ± 4 °C) with 60 % water-holding capacity throughout the incubation period. After desired intervals of incubation, soil samples were extracted in distilled water for estimation of enzyme activities. Similar model was used earlier by (Singaram and Kamalakumari 2010; Mohiddin et al. 2010).

In order to determine cellulase enzyme activity in soils, the method employed for the assay of cellulase was developed by Cole (1977) and followed by Tu (1981a, b). The soil samples were transferred to 100 ml Erlenmeyer flasks and were treated with 1 ml of toluene to arrest the enzyme activity. After 15 min, 10 ml of carboxy methyl cellulose (CMC) 1 % was used as a substrate followed by 10 ml of acetate buffer (pH 5.9) and incubated for 24 h to determine the reducing sugar content in the filtrate (Deng and Tabatabai 1994). In another experiment, cellulase activity was determined at 10, 20, 30 and 40 days of soil incubation. Testing samples were passed through Whatman No. 1 filter paper and the filtrate was assayed for the amount of glucose by the Nelson method (1944) in a Spectronic 20 D spectrophotometer.

The method employed for assay of invertase was developed by Cole (1977) and followed by (Tu 1981a, b). The soil samples were transferred to 100 ml Erlenmeyer flasks and treated with 1 ml toluene to arrest the enzyme activity. After 15 min, 6 ml of 18 mM sucrose was added to the soil samples and incubated for 24 and 48 h; the testing samples were passed through Whatman No. 1 filter paper and the filtrate was assayed for the amount of glucose by the Nelson method (1944) in a Spectronic 20 D spectrophotometer.

The method employed for the assay of amylase was developed by Cole (1977) and followed by Tu (1981a, b). The soil samples were transferred to 100 ml Erlenmeyer flasks and treated with 1 ml toluene to arrest the enzyme activity. After 15 min, 6 ml of 0.2 M of acetate phosphate buffer (5.5 pH) containing 2 % starch was added to each of the testing samples and closed with cotton plugs. After 24 and 72 h of incubation, the testing samples were made up to a volume of 50 ml with sterile distilled water and passed through Whatman No. 1 filter paper and the filtrate was assayed for the amount of glucose by Nelson’s method (1944) in a Spectronic 20 D spectrophotometer.

### Statistical analysis

The concentration of the cellulase, invertase and amylase was calculated based on soil weight (oven dried). Data were analyzed using one-way ANOVA, and the differences contrasted using Duncan’s multiple range test (DMRT) (Megharaj et al. 1999; Mohiddin et al. 2011). All statistical analyses were performed at *P* ≤ 0.05 using SPSS statistical software package.

## Results and discussion

The black and red clay soils are predominantly used for the cultivation of groundnut (*Arachis hypogaea* L.) in the Anantapur district of Andhra Pradesh, India. The major constraints in the groundnut crop are insects and fungi pests. For this reason, pesticides are frequently used for crop protection. Continuous and indiscriminate use of these pesticides causes a major risk of soil health. Hence, these soils were selected to study the effect of insecticides on enzyme activities. In general, the organic matter content is high in black soil. Therefore, the biological activity was also pronounced more in black soil than in red soil under the influence of insecticides. There have been many reports of the effects of pesticides on soil enzyme activities (Anonymous 2011; Loganathan et al. 2002) and it has been observed that the responses of soil enzymes to different pesticides are not the same. As a new pesticide, unfortunately, there is no information available regarding the influence of flubendiamide and spinosad on soil enzyme activities. Of course, when the flubendiamide and spinosad concentration was increased, the potential hazard to soil would increase. Soil enzyme activities are more sensitive to the environment. They reflect the soil quality more quickly and directly (Srinivasulu et al. 2012).

**Fig. 1:**
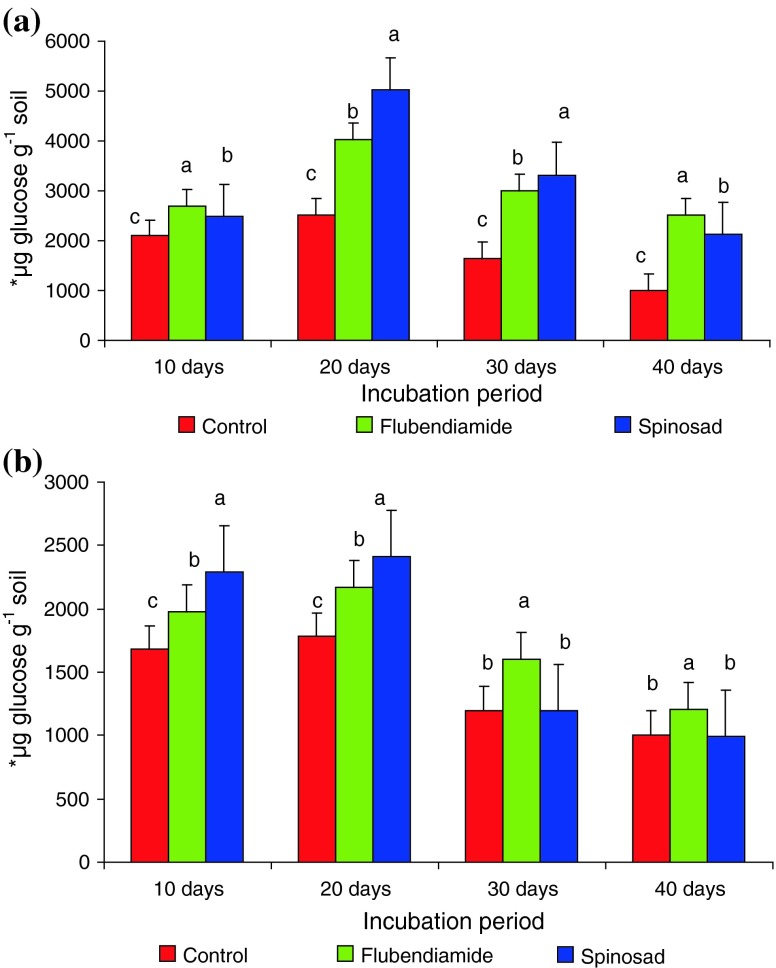
Influence of flubendiamide and spinosad on cellulase*activity in **a** black clay and **b** red clay soil at 2.5 kg ha^−1^. *μg glucose per gram soil formed after 24 h incubation with Carboxy methyl cellulose (CMC). The values are the mean ± SE for each incubation period, are not significantly different (*P* ≤ 0.05) from each other according to Duncan’s multiple range (DMR) test

Since enzyme activity has been considered as a very sensitive indicator, any disturbance due to biotic or environmental stresses in the soil ecosystem may affect soil biological properties. Our analysis revealed that cellulase activity was significantly increased from 0.1 to 2.5 kg ha^−1^ whereas the activity was decreased at higher concentrations (5.0–10.0 kg ha^−1^) of pesticides in both soils (Table 2). The cellulase activity was significantly enhanced at 2.5 kg ha^−1^ level in both soils for flubendiamide and spinosad and showed individual increments of cellulase activity ranging from a low increase 15–29, 11–19 and 2–18, 12–36 % in comparison to control (Table 2). The stimulatory concentration (2.5 kg ha^−1^) induces the highest cellulase activity after 20, 30 and 40 days of incubation in black clay soils (Fig. 1a) with flubendiamide and spinosad when compared to control. Whereas in red clay soil a similar trend was followed by flubendiamide, induces the highest cellulase activity after 20, 30 and 40 days of incubation but spinosad showed a variable pattern was observed at 30 and 40 days, the cellulase enzyme activity remained same with control (Fig. 1b). The relatively low activity of cellulase might result from the toxic effect of flubendiamide and spinosad on soil microorganisms, which in turn produces cellulase. The inhibition of cellulase activity by flubendiamide and spinosad could be attributed to the properties of flubendiamide and spinosad. Similar type of reports were identified by (Ramudu et al. 2011; Mohiddin et al. 2010) chlorothalonil and propiconazole, imidacloprid, and acephate. Similar observations were made by Katayama and Kuwatsuka (1991) and Jaya Madhuri and Rangaswamy (2002) on the cellulase activity. Analogous report was obtained by Ismail et al. (1996a, b) on application of metolachor to Malaysian soil. Gigliotti et al. (1998) also reported that bensulfurn methyl at 16 and 160 µg/g inhibited cellulase activity in soil samples. In a diverse study made by Gherbawy and Abdelzaher (1999), alteration in the activity of cellulase by metalaxyl was marked in pure fungal cultures. Similar results were obtained by Arinz and Yubedee (2000) that kelthane and fenvalerate caused inhibition to enzyme activity.

**Table 2:** Activity of cellulase under the impact of different concentrations of flubendiamide and spinosad in black and red clay soils for 24 h after 10 days

Concentration of insecticides (kg ha^−1^)	Black clay soil		Red clay soil	
	Flubendiamide	Spinosad	Flubendiamide	Spinosad
	24 h	24 h	24 h	24 h
0.0	2,090 ± 5.773 c (100)	2,090 ± 5.773 c (100)	1,680 ± 11.547 c (100)	1,680 ± 11.547 d (100)
1.0	2,400 ± 57.735 b (115)	2,330 ± 17.320 b (134)	1,720 ± 5.773 b (102)	1,880 ± 11.547 c (112)
2.5	2,700 ± 2.886 a (129)	2,490 ± 2.886 a (172)	1,980 ± 0.577 a (118)	2,290 ± 4.041 a (136)
5.0	1,700 ± 17.320 d (81)	1,350 ± 5.773 d (134)	1,400 ± 17.320 d (83.3)	2,200 ± 57.735 b (130)
7.5	1,390 ± 4.041 e (66)	1,190 ± 0.577 f (128)	1,090 ± 2.309 e (65)	1,200 ± 25.980 e (71)
10.0	1,100 ± 5.773 f (52)	1,250 ± 3.464 e (60)	990 ± 1.732 f (97)	1,120 ± 14.433 f (67)

μg glucose per gram soil formed after 24 h of incubation with 1 % carboxy methyl cellulose (CMC)

Each column is mean ± SE for six concentrations in each group; columns not sharing a common letter (a, b, c, d, e and f) differ significantly with each other (*P* ≤ 0.05; DMRT)

**Fig. 2:**
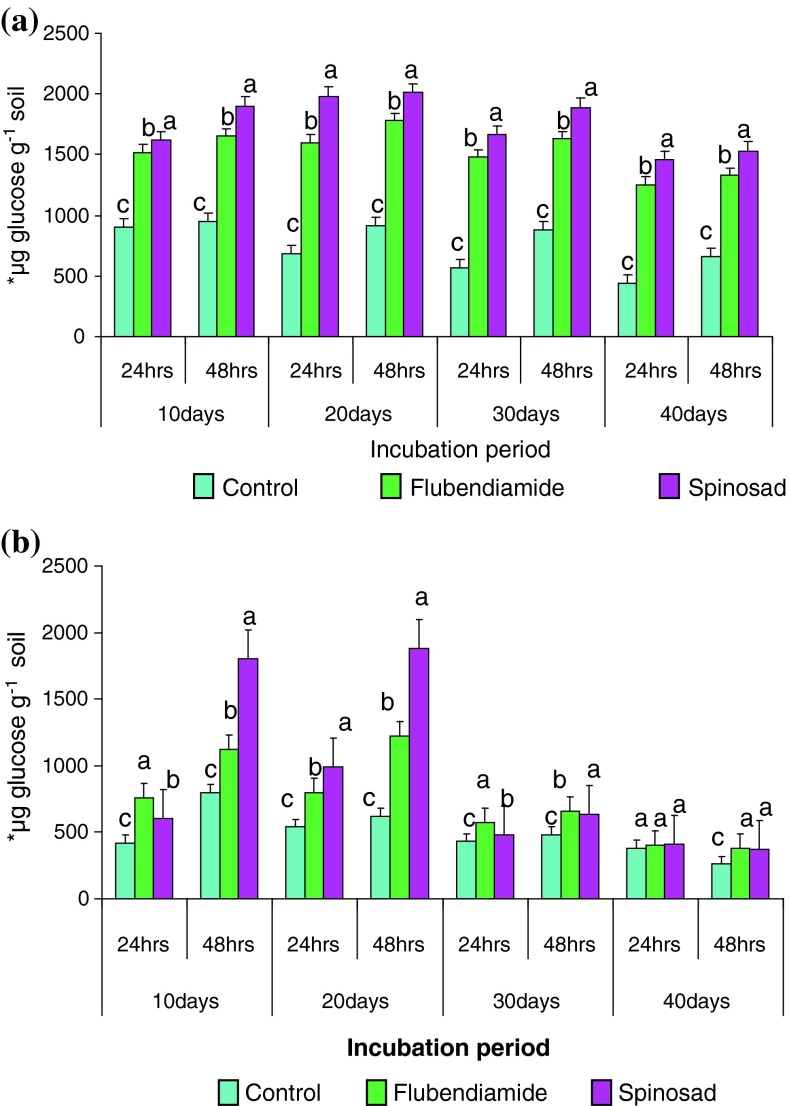
Influence of flubendiamide and spinosad on invertase*activity in **a** black clay and **b** red clay soil at 2.5 kg ha^−1^. *μg glucose per gram soil formed after 24 and 48 h incubation with 18 Mm sucrose. The values are the mean ± SE for each incubation period, are not significantly different (*P* ≤ 0.05) from each other according to Duncan’s multiple range (DMR) test

Invertase activity was depressed in flubendiamide and spinosad treated soils throughout the experiment when compared to the controls in both soils incubated for 10 days (Tables 3, 4) The maximum activity was observed at 2.5 kg ha^−1^ (stimulatory) for flubendiamide and spinosad showed individual increments of invertase activity ranged from a low increase 2–69, 1–74 % and 0–80, 87–100 % for black clay soil and for red clay soil, 43–81, 27–40 % and 38–43, 65–125 % received 2.5 kg ha^−1^ respectively in comparison to control at 24 and for 48 h (Tables 3, 4). The results reveal that invertase enzyme is rather sensitive to flubendiamide and spinosad. Figure 2 showed the variation of invertase activity after flubendiamide and spinosad application. Although enzyme activities of samples were lower than that of control, significant differences (*P* ≤ 0.05) were found among the enzyme activities between treated soil samples and the control (Tables 3, 4). With the increase in incubation periods, the stimulated enzyme activities were also increased up to 20-days further increase in the incubation decrease in the enzyme activity was noticed (Fig. 2a, b). Our results appeared to be consistent with previous reports, in which it is demonstrated that pesticides stimulated invertase activity of soils (Ramudu et al. 2011; Sannino and Gianfreda 2001; Srinivasulu and Rangaswamy 2006). Rate of invertase activity followed the same trend of initial stimulation followed by inhibition as reported by Rangaswamy and Venkateswarlu (1992). On the contrary, Tu (1995) affirmed initial inhibition followed by recovery with five insecticides in sandy loam soil.

**Table 3:** Activity of invertase under the impact of different concentrations of flubendiamide and spinosad in black clay soil for 24 and 48 h after 10 days

Concentration of insecticides (kg ha^−1^)	Flubendiamide		Spinosad	
	24 h	48 h	24 h	48 h
0.0	900 ± 0.577 c (100)	950 ± 2.309 e (100)	900 ± 0.577 c (100)	950 ± 2.309 e (100)
1.0	915 ± 2.886 b (102)	960 ± 5.773 d (101)	900 ± 0.577 b (100)	1,780 ± 11.547 c (187)
2.5	1,520 ± 5.773 a (169)	1,650 ± 4.618 a (174)	1,620 ± 4.618 a (180)	1,900 ± 17.320 a (200)
5.0	800 ± 5.773 d (89)	1,310 ± 1.732 b (138)	600 ± 0.577 d (67)	1,850 ± 5.773 b (195)
7.5	750 ± 3.464 e (83)	980 ± 11.547 c (103)	440 ± 23.094 e (49)	1,060 ± 3.464 d (111)
10.0	700 ± 1.732 f (78)	800 ± 5.773 f (84)	400 ± 2.886 f (44)	900 ± 0.577 f (95)

μg glucose per gram soil formed after 24 h of incubation with 18 Mm sucrose

Each column is mean ± SE for six concentrations in each group; columns not sharing a common letter (a, b, c, d, e and f) differ significantly with each other (*P* ≤ 0.05; DMRT)

**Table 4:** Activity of invertase under the impact of different concentrations of flubendiamide and spinosad in red clay soil for 24 and 48 h after 10 days

Concentration of insecticides (kg ha^−1^)	Flubendiamide		Spinosad	
	24 h	48 h	24 h	48 h
0.0	420 ± 4.618 f (100)	800 ± 8.660 d (100)	420 ± 4.618 d (100)	800 ± 5.773 f (100)
1.0	600 ± 0.577 d (143)	1,020 ± 4.618 b (127)	580 ± 11.547 b (138)	1,320 ± 4.041 d (165)
2.5	760 ± 4.041 a (181)	1,120 ± 11.547 a (140)	600 ± 0.577 a (143)	1,800 ± 0.577 a (225)
5.0	700 ± 1.732 b (166)	920 ± 4.618 c (115)	589 ± 11.547 b (140)	1,720 ± 11.547 c (215)
7.5	650 ± 28.867 c (155)	780 ± 11.547 e (97)	490 ± 2.886 c (117)	1,620 ± 5.773 b (202)
10.0	620 ± 11.547 e (148)	670 ± 17.320 f (84)	350 ± 10.392 e (83)	1,300 ± 2.886 e (162)

μg glucose per gram soil formed after 24 h of incubation with 18 Mm sucrose

Each column is mean ± SE for six concentrations in each group; columns not sharing a common letter (a, b, c, d, e and f) differ significantly with each other (*P* ≤ 0.05; DMRT)

**Fig. 3:**
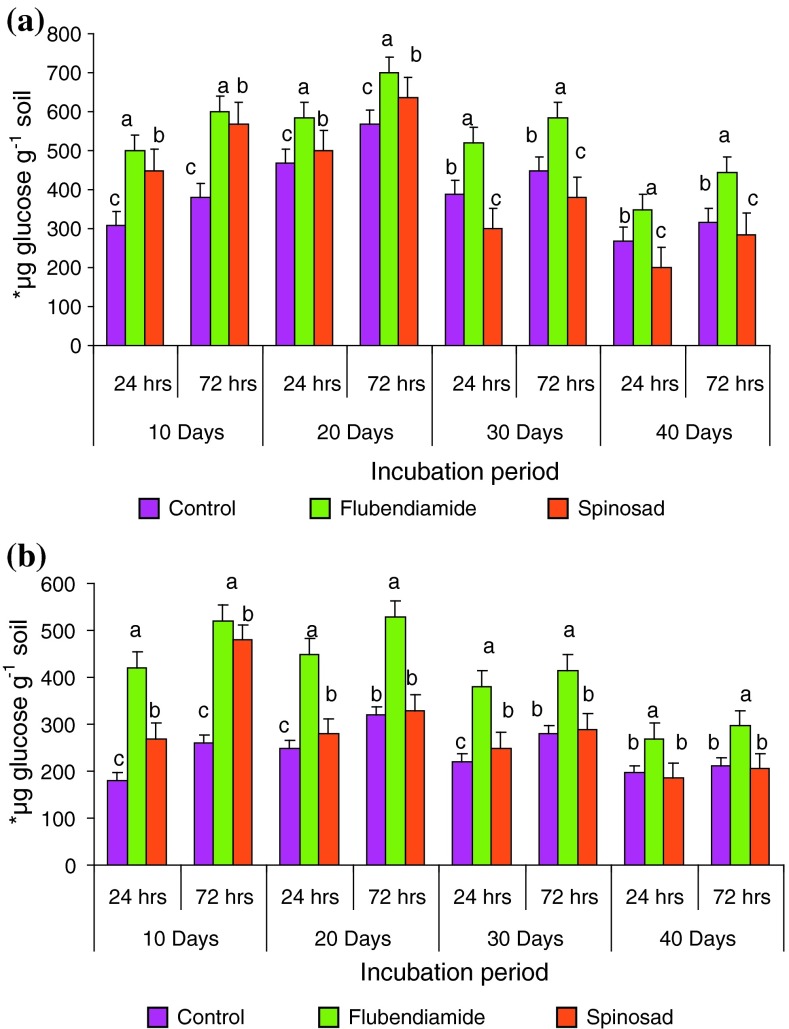
Influence of flubendiamide and spinosad on amylase*activity in **a** black clay and **b** red clay soil at 2.5 kg ha^−1^. *μg glucose per gram soil formed after 24 and 72 h incubation with 2 % starch. The values are the mean ± SE for each incubation period, are not significantly different (*P* ≤ 0.05) from each other according to Duncan’s multiple range (DMR) test

Amylase activity (Tables 5, 6) showed a variable pattern in response to different insecticide concentration after 10 days of incubation. Amylase activity increased under lower doses and decreased under higher doses compared to the controls in black and red clay soils. The maximum activity was observed at 2.5 kg ha^−1^ (stimulatory) for flubendiamide, spinosad. Amylase activity showed an individual increment of 35–61, 29–58, 0–45, 8–50 %, in black clay soil and 61–133, 31–100, 39–50, 15–85 %, in comparison to control at 24 and for 72 h received 2.5 and 5.0 kg ha^−1^ respectively in red clay soil. With the increase in incubation periods, the stimulated enzyme activities were also increased up to 20-days further increase in the incubation decrease in the enzyme activity was noticed (Fig. 3). Our results were in contrast with the several researcher works (Srinivasulu and Rangaswamy 2006; Mohiddin et al. 2010; Tu 1981a, b, 1982, 1988), triazophos, a phosphorothioate triazole is stimulated for amylase at 5 and 10 mg/kg incubated for 3 days in an organic soil. As per the observation made by the Prasad and Mathur (1983) the amylase activity increased during germination in both control, and Cuman treated seeds at 0.25, 0.5, 0.75 and 1 % respectively. Interaction effects on soil enzyme activities, including amylase activity received least attention. There were only isolated reports on interaction effects between two chemical compounds in axenic culture studies with algae, cyanobacteria and fungi (Megharaj et al. 1989; Stratton and Corke 1982a, b). Kennedy and Arathan (2002) reported that application of carbofuran at 1 and 1.5 kg ha^−1^ significantly reduced the activity of soil enzymes, viz., alpha -amylase, beta -glucosidase, cellulase, urease and phosphatase up to 30 days after carbofuran application. However, application of carbofuran at the recommended level (0.5 kg a.i. ha^−1^) had no significant effect upon the activity of soil enzymes, which are biologically significant as they play an important role not only in the soil chemical and biological properties but also affect the nutrient availability to plants. Rate of amylase activity followed the same trend of initial stimulation followed by inhibition as reported by Rangaswamy and Venkateswarlu (1992) and Vijay Gundi et al. (2007). Thus, far, no information has been available regarding the influence of flubendiamide and spinosad on these soil enzyme activities. At the same time, much more should be done to understand the influence of flubendiamide and spinosad on soil enzymes clearly. Hence further investigation is needed to evaluate the influence of insecticides on the enzyme activities in agricultural soils which are important and affect nutrient cycling and fertility of soils.

**Table 5:** Activity of amylase under the impact of different concentrations of flubendiamide and spinosad in red clay soil for 24 and 72 h after 10 days

Concentration of insecticides (kg ha^−1^)	Red clay soil			
	Flubendiamide		Spinosad	
	24 h	72 h	24 h	72 h
0.0	180 ± 2.886 d (100)	260 ± 2.886 d (100)	180 ± 2.886 d (100)	260 ± 2.886 c (100)
1.0	290 ± 2.309 b (161)	340 ± 5.773 b (131)	250 ± 5.773 b (139)	300 ± 0.577 b (115)
2.5	420 ± 1.732 a (233)	520 ± 0.577 a (200)	270 ± 1.732 a (150)	480 ± 2.309 a (185)
5.0	200 ± 2.309 c (111)	280 ± 17.320 c (108)	200 ± 0.577 c (111)	300 ± 2.309 b (115)
7.5	160 ± 2.309 e (89)	200 ± 0.577 e (77)	205 ± 2.886 c (114)	265 ± 2.886 c (102)
10.0	120 ± 5.773 f (67)	180 ± 11.547 f (69)	180 ± 2.886 d (100)	250 ± 5.773 d (96)

μg glucose per gram soil formed after 24 and 72 h of incubation with 2 % starch

Each column is mean ± SE for six concentrations in each group; columns not sharing a common letter (a, b, c, d and e) differ significantly with each other (*P* ≤ 0.05; DMRT)

**Table 6:** Activity of amylase under the impact of different concentrations of flubendiamide and spinosad in black clay soil for 24 and 72 h after 10 days

Concentration of insecticides (kg ha^−1^)	Black clay soil			
	Flubendiamide		Spinosad	
	24 h	72 h	24 h	72 h
0.0	310 ± 5.773 d (100)	380 ± 2.886 e (100)	310 ± 5.773 b (100)	380 ± 2.886 c (100)
1.0	420 ± 0.577 b (135)	490 ± 5.773 c (129)	310 ± 11.547 b (100)	450 ± 17.320 d (118)
2.5	500 ± 8.660 a (161)	600 ± 11.547 a (158)	450 ± 5.773 a (145)	570 ± 1.154 a (150)
5.0	450 ± 2.886 b (145)	520 ± 1.154 b (137)	300 ± 0.577 b (97)	400 ± 5.773 b (105)
7.5	350 ± 17.320 c (113)	460 ± 2.309 d (121)	250 ± 2.886 c (81)	305 ± 1.154 e (80)
10.0	210 ± 5.773 e (67)	320 ± 5.773 f (84)	240 ± 5.773 c (77)	280 ± 1.154 f (74)

μg glucose per gram soil formed after 24 and 72 h of incubation with 2 % starch

Each column is mean ± SE for six concentrations in each group; columns not sharing a common letter (a, b, c, d and e) differ significantly with each other (*P* ≤ 0.05; DMRT)

## Conclusions

Results from this study indicated that the cellulase enzyme activity was profoundly increased up to 2.5 kg ha^−1^ where as at higher concentrations (5.0–10.0 kg ha^−1^) of pesticide concentration the enzyme activity were dramatically decreased in both the soils except spinosad at 5.0 kg ha^−1^ in red clay soil. Invertase enzyme activity was decreased from 5.0–10.0 kg ha^−1^ level when compared to control in black clay soil where as the enzyme activity at 1.0–2.5 kg ha^−1^ level. In red clay soil the invertase enzyme activity was stimulated up to 10.0 kg ha^−1^ when compared to control except fubendiamide for 48 h and for spinosad for 24 h at 7.5–10.0 kg ha^−1^. Amylase enzyme activity showed a stimulatory activity up to 5.0 kg ha^−1^ further increase in the pesticide concentration repression in the enzyme activity was noticed in both soils. Overall soil enzymes were affected by the application of flubendiamide and spinosad at higher concentrations (5.0–10.0 kg ha^−1^). However, as an important agent for the control of plant pathogens, flubendiamide and spinosad is often used at rates much greater than the recommended dosage.

Overall, flubendiamide and spinosad at a normal field dose (1.0–2.5 kg ha^−1^) would not pose a threat to soil enzymes among them spinosad is more effective than flubendiamide in inducing the cellulase and invertase with exception of amylase enzyme activities at normal field rates (1.0–2.5 kg ha^−1^). When flubendiamide and spinosad concentration was increased (5.0–10.0 kg ha^−1^), however, the threat to soil, cellulase, invertase and amylase increased. A very few reports are available on the influence of insecticides on these enzyme activities cellulase, invertase, and amylase.
